# A technique for intra-procedural blood velocity quantitation using time-resolved 2D digital subtraction angiography

**DOI:** 10.1186/s42155-020-00199-y

**Published:** 2021-01-07

**Authors:** Carson Hoffman, Sarvesh Periyasamy, Colin Longhurst, Rafael Medero, Alejandro Roldan-Alzate, Michael A. Speidel, Paul F. Laeseke

**Affiliations:** 1 Department of Medical Physics, University of Wisconsin – Madison, 1111 Highland Ave, Madison, WI 53705 USA; 2 Department of Biomedical Engineering, University of Wisconsin – Madison, 1111 Highland Ave, Madison, WI 53705 USA; 3 Department of Biostatistics and Medical Informatics, University of Wisconsin – Madison, 1111 Highland Ave, Madison, WI 53705 USA; 4 Department of Mechanical Engineering, University of Wisconsin – Madison, 1111 Highland Ave, Madison, WI 53705 USA; 5 Department of Radiology, University of Wisconsin – Madison, 1111 Highland Ave, Madison, WI 53705 USA; 6 Section of Interventional Radiology, Department of Radiology, University of Wisconsin – Madison, 600 Highland Ave, Madison, WI 53792 USA

**Keywords:** Digital subtraction angiography, Quantitative, Arterial velocity, Time-attenuation curve

## Abstract

**Background:**

2D digital subtraction angiography (DSA) is utilized qualitatively to assess blood velocity changes that occur during arterial interventions. Quantitative angiographic metrics, such as blood velocity, could be used to standardize endpoints during angiographic interventions.

**Purpose:**

To assess the accuracy and precision of a quantitative 2D DSA (qDSA) technique and to determine its feasibility for in vivo measurements of blood velocity.

**Materials and methods:**

A quantitative DSA technique was developed to calculate intra-procedural blood velocity. In vitro validation was performed by comparing velocities from the qDSA method and an ultrasonic flow probe in a bifurcation phantom. Parameters of interest included baseline flow rate, contrast injection rate, projection angle, and magnification. In vivo qDSA analysis was completed in five different branches of the abdominal aorta in two 50 kg swine and compared to 4D Flow MRI. Linear regression, Bland-Altman, Pearson’s correlation coefficient and chi squared tests were used to assess the accuracy and precision of the technique.

**Results:**

In vitro validation showed strong correlation between qDSA and flow probe velocities over a range of contrast injection and baseline flow rates (slope = 1.012, 95% CI [0.989,1.035], Pearson’s r = 0.996, *p* < .0001). The application of projection angle and magnification corrections decreased variance to less than 5% the average baseline velocity (*p* = 0.999 and *p* = 0.956, respectively). In vivo validation showed strong correlation with a small bias between qDSA and 4D Flow MRI velocities for all five abdominopelvic arterial vessels of interest (slope = 1.01, Pearson’s r = 0.880, p = <.01, Bias = 0.117 cm/s).

**Conclusion:**

The proposed method allows for accurate and precise calculation of blood velocities, in near real-time, from time resolved 2D DSAs.

**Supplementary Information:**

The online version contains supplementary material available at 10.1186/s42155-020-00199-y.

## Introduction

Angiographic procedures such as angioplasty, stent placement and transarterial embolization (TAE) are largely qualitative, relying on subjective, visual assessment of digital subtraction angiography (DSA) images to diagnose pathology, determine procedural endpoints, and evaluate treatment efficacy. Angiographic assessment using DSA depends on factors such as observer experience and perceptual bias, both of which have been previously shown to be a significant source of interpretive error in radiology (Lee et al. 2013). This leads to a high degree of interobserver variability and a decrease in reproducibility (Koelemay et al. 2001; Paul et al. 1999; de Vries et al. 1984). Procedural outcomes are subsequently affected, including variable success rates in balloon angioplasty in peripheral arterial disease and poor correlation of tumoral perfusion changes with subjective treatment endpoints during TAE (Lewandowski et al. 2007; Gardiner et al. 2004). Quantitative angiographic metrics may be beneficial in standardizing angiographic body interventions, ultimately improving their safety and efficacy.

Quantitative angiographic techniques, including quantitative color-coded DSA, 4D DSA, 4D Flow MR and 4D transcatheter perfusion, can provide data on hemodynamic parameters (e.g. time of arrival, flow, velocity) (Meram et al. 2019; Shaughnessy et al. 2018; Wu et al. 2018; Motoyama et al. 2017; Nett et al. 2012; Frydrychowicz et al. 2011; Wang et al. 2010; Gu et al. 2005). The application of MRI techniques for real-time guidance is limited by the need for additional specialized equipment and suites. 4D DSA requires rotational scans with relatively long data acquisition times, practically limiting the number of 4D DSAs that can be acquired during any given procedure. Quantitative color-coded DSA can provide color-coded vessel displays based on time of arrival (TOA) or time to peak (TTP) during intravascular procedures (Strother et al. 2010; Lin et al. 2018; Iwakoshi et al. 2019) but is highly susceptible to image artifact and variation in injection parameters (Shpilfoygel et al. 2000; Ionita et al. 2014; Kennedy et al. 2010).

DSA is the gold-standard imaging method for guiding and assessing intravascular procedures. A robust quantitative angiography technique that could provide blood velocities utilizing DSA would be minimally intrusive to the procedural workflow and would provide a quantitative complement to current assessment methods. For example, DSA in commonly used in the treatment of peripheral arterial disease for balloon angioplasty and stent placement (Gardiner et al. 2004). The pre- and post- procedural imaging series can provide clear quantitative structural changes, while hemodynamic changes are subjectively assessed through the interpretation of contrast dynamics. While color-coded DSA has been used to provide quantitative temporal information such as TOA or TTP (Strother et al. 2010; Iwakoshi et al. 2019), these techniques rely on the selection of a single point from the contrast curve and does not provide true arterial velocity or flow.

The purpose of this study was to assess the accuracy, precision, and feasibility of a quantitative angiography technique which extracts blood velocity from time-resolved 2D DSA (qDSA) sequences, utilizing the contrast dynamics over the entire injection period.

## Materials & methods

All studies were performed with approval from the institutional animal care and use committee and complied with National Research Council guidelines.

### Quantitative angiography method

**Fig. 1:**
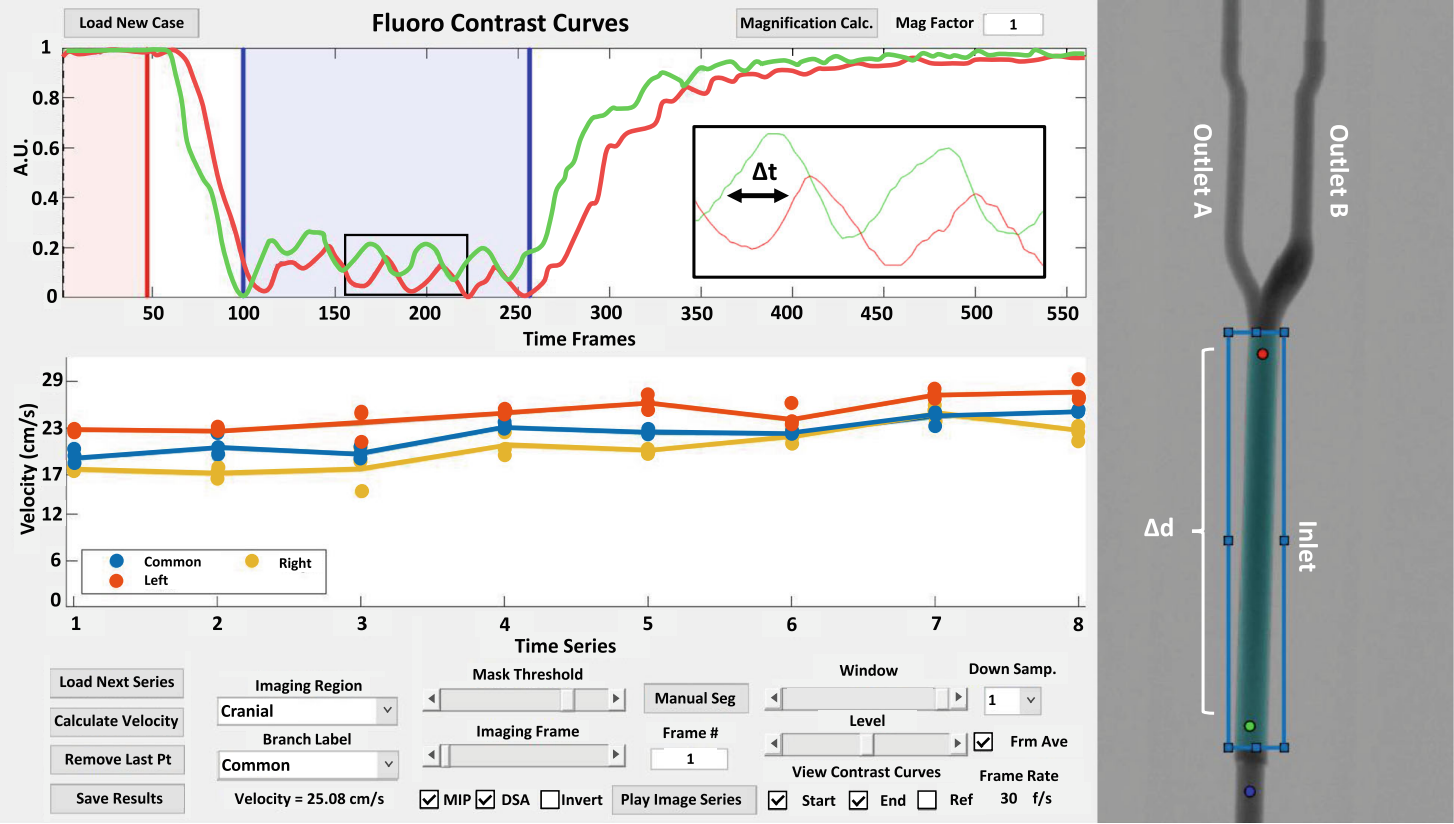
qDSA calculations were completed with the custom built post processing tool in MATLAB. Vessel start and end points are selected by moving the red and green points on the acquired image. Temporal windowing is adjusted by changing the blue shaded region overlaid on the time attenuation curves. The spatial information and temporal information used for velocity calculation are derived from the Δd and Δt. Visualization of the acquired image series can be completed using the tool’s built in functions

The proposed quantitative angiography method uses the inherent cardiac pulsatility of arterial flow in addition to spatial information within an artery to compute blood velocity. Pulsatility of arterial blood flow during a contrast injection results in an oscillating contrast signal in the time-attenuation curve (TAC) of a given pixel in the DSA image sequence. This oscillation of signal, referred to as contrast pulsatility, represents a trackable marker of blood flow. Given two pixels along a vessel separated by distance ***d***, the TACs from the two pixels will have similar pulsatile signals, offset by some temporal shift ***t*** (Fig. 1). This temporal shift corresponds to the time a contrast bolus travels through a vessel. A shifted-least squares approach is employed to calculate the temporal shift (Wu et al. 2018). Distances and temporal shifts can be computed for pairs of points along the vessel centerline to improve statistical power and results in a spatially averaged blood velocity ***v***.

Blood velocities were calculated using a custom-built MATLAB tool (Fig. 1). The tool imports a DSA sequence and allows the user to choose a vessel of interest by selecting a proximal and distal point along the vessel. The vessel can then be manually or automatically segmented, after which the tool automatically determines the centerline. The user may view and window the time-intensity curves from the two selected points in order to select the region of strongest pulsatility. A correction for geometric magnification may be calculated and input into the tool by measuring a structure in the image and dividing by its known length (Magnification = Image Length / Object Length). The tool subsequently calculates an apparent blood velocity then multiplies by the magnification factor to convert it to a true velocity. Vessel foreshortening effects can arise for a vessel that is not perpendicular to the projection angle. Projection angle can be corrected for by dividing the apparent velocity by the cosine of projection angle offset (Velocity = apparent velocity / cos (projection angle)). Alternatively, a 3D angiogram can be utilized to select an optimal 2D projection that minimizes vessel foreshortening.

### Phantom study

In vitro validation of the qDSA method was performed in a silicone vascular bifurcation phantom (Shelley Medical Imaging Technologies, Ontario, Canada) to assess the algorithm in a controlled environment using different image acquisition parameters. DSA acquisitions were performed in triplicates for each experimental setup using an Artis zee x-ray system (Siemens Healthineers, Forcheim, Germany) and angiographic injector (Nemoto, Tokyo, Japan). A pulsatile displacement pump (BDC Laboratories, Wheat Ridge, CO), filled with water, was connected to the phantom in order to produce a physiologic flow profile similar to a cardiac cycle at a rate of 60 beats per minute. Average inlet velocity was calculated during all DSA acquisitions using a non-intrusive ultrasonic flow sensor measurement (Transonic, Ithaca, NY) and dividing by the known tube diameters. Imaging parameters of interest included baseline flow rate, contrast injection rate, projection angle, and magnification. To investigate these parameters, we varied the pump velocity, injector rate, gantry rotation and table height respectively. A guidewire, with a 3 cm long radiopaque tip, was navigated into the phantom and was used to correct for magnification. A complete list of imaging parameters for all experiments are included in Table 1.

**Table 1:** In Vitro Imaging Parameters

Baseline Flow	Pump Flow (ml/s)	Injection Rate (ml/s)	SID (cm)	Projection Angle	Injection Time (s)
	**6.8**	**1.5–3**	120	0	8
	**12.2**	**1.5–3**	120	0	8
	**16.8**	**1.5–3**	120	0	8
	**22.1**	**1.5–3**	120	0	8
	**27.2**	**1.5–3**	120	0	8
**Projection Angle**					
	5.2	2	120	**0**	10
	5.2	2	120	**5**	10
	5.2	2	120	**10**	10
	5.2	2	120	**20**	10
	5.2	2	120	**30**	10
**Magnification**					
	7.4	2.5	**90**	0	6
	7.4	2.5	**100**	0	6
	7.4	2.5	**110**	0	6
	7.4	2.5	**120**	0	6

Experimental parameters used to evaluate baseline flow rate, injection rate, projection angle, and magnification are present in the table. The bolded values are the parameter that is being varied during each experiment

### In vivo study

The in vivo validation of the qDSA method was performed in a porcine model. Female domestic swine (*n* = 2, 50 kg, approximately 3–4 months of age) were sedated with intramuscular tiletamine hydrochloride/zolazepam hydrochloride (7 mg/kg; Telazol; Fort Dodge Animal Health, Fort Dodge, IA) and xylazine hydrochloride (2.2 mg/kg; Xyla-Ject; Phoenix, St. Joseph, MO). Anesthesia was maintained with inhaled 1.0%–2.0% isoflurane (Halocarbon, River Edge, NJ). An auricular vein was cannulated for administration of intravenous fluids. First, the animals underwent 4D Flow MRI (3 T Signa MRI scanner, GE Healthcare, Waukesha, WI) using a radially undersampled sequence, PC VIPR (Johnson and Markl 2010). The animal was then transferred to the angiography suite. The right femoral artery was accessed and a 5Fr angled Glide Catheter (Merit Medical, Salt Lake City, UT) was used to select arteries of interest (left iliac, left and right renals, common hepatic, splenic). A guidewire, with a 3 cm radiopaque tip, was navigated into the vessel of interest and a fluoroscopic image was acquired prior to DSA acquisition for geometric magnification corrections. Projection angles for 2D DSA acquisitions were selected to minimize the effect of vessel foreshortening. Triplicate DSAs were acquired in each artery with a breath hold at end expiration. A complete list of imaging parameters used for MR and DSA acquisitions can be seen in Table 2. Velocities were calculated and compared between the MR and DSA techniques. A semi-automated workflow for the 4D flow MRI analysis was developed in MATLAB 2018a. Angiograms were segmented using an adaptive region growing technique on the complex difference data. A 3D centerline path was generated to aid in the automatic placement of cross-sectional planes. Automatic segmentation of vessels was completed using a local thresholding technique and reported velocities were an average of all points along the vessel centerline. In order to compare velocities on MRI to those on DSA, which are acquired in the presence of intra-arterial contrast injection, velocities calculated on MRI were adjusted by adding velocity from an injection equivalent to that performed during acquisition of the DSAs (programed injection rate (2 ml/s) divided by the 4D flow MRI vessel area).

**Table 2:** In Vivo Imaging Parameters

DSA Swine Scan	Injection Rate (ml/s)	Injection Time (s)	Projection Angle	Frame Rate (fps)
All Vessels	2	12	0	30
**MRI Swine Scan**	Spatial Res. (mm)	Scan Time (min)	VENC (cm/s)	Time Frames
All Vessels	1 × 1 × 1	18	100	14

In vivo scan parameters for qDSA and 4D Flow MRI. Identical scans were used for all vessel locations, including: Iliac, right renal, left renal, common hepatic, splenic. MRI time frames is the number of reconstructed volumes for a cardiac cycle. The velocity encoding (VENC) was set to 100 cm/s in order to capture velocities from all abdominal vessels in a single scan

### Statistical analysis

To assess the correlation between the external flow probe and the velocities calculated from the qDSA technique in the phantom study, linear regression was used. Associated model *p*-values, 95% confidence intervals and Pearson’s correlation coefficient, *r,* were all calculated following model estimation. Linear regression and Bland-Altman analysis were used to assess the calibration between qDSA velocity and 4D flow MRI in the in-vivo study. Right-tailed chi-square tests were used to assess if the observed variances of the phantom qDSA measurements across different magnifications and angle corrections were less than 5% of the mean in the associated flow probe. A desired variance of 5% of the mean velocity from the ultrasonic flow probe was set as the upper limit for the projection angle and magnification correction techniques. This was used to establish the precision of our tool in cases where corrective factors may be needed. For this study, a *p*-value < 0.05 was considered statistically significant. All statistical analyses were done using R (V 3.6.2, R Core Team, 2019).

## Results

### Phantom study

Quantitative velocities were computed using the proposed custom-built MATLAB tool. The tool was able to successfully load DSA image series, select vessels of interest, complete vessel segmentation, allow temporal windowing, and compute velocities for all cases. Magnification corrections from a reference object were computed and implemented into the velocity calculations for all DSA acquisitions. All computations were completed within 2–3 min of acquisition during experiments. The physiologic pulsatile cardiac waveforms were inspected using the non-intrusive ultrasonic flow probe prior to and during DSA acquisition. The waveforms maintained their pulsatility for all pump and injection rates. Adequate contrast mixing was seen in all DSA acquisitions completely filling the vessel and maintaining a pulsatile nature.

**Fig. 2:**
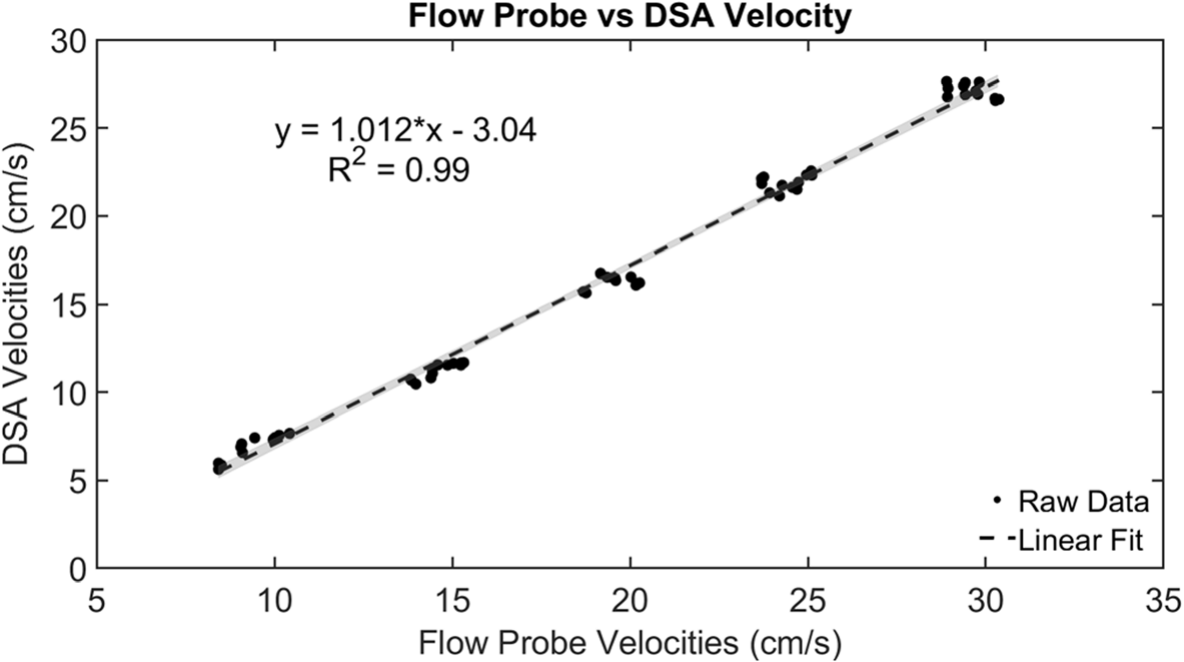
Linear regression analysis between the flow probe velocities and the calculated qDSA velocities. The shaded region represents the 95% confidence interval. The data were found to be strongly correlated (*r =* 0.996, *p* < .0001)

The pulsatile pump’s average and peak velocities were measured prior to the bifurcation and ranged from 8 to 30 cm/s and 13–55 cm/s respectively. Linear regression between the calibrated external flow probe and velocities calculated from our qDSA technique is shown in Fig. 2. The linear regression equation was: V_DSA_ = 1.012*V_US_ – 3.043, where V_DSA_ is the velocity calculated from qDSA and V_US_ is the velocity measured with the ultrasound flow probe. A strong correlation between the variables was observed (*r =* 0.996, *p* < .0001). The 95% confidence interval (CI) for the slope was [0.989,1.035] and the 95% CI for the intercept was [− 3.52, − 2.565].

**Fig. 3:**
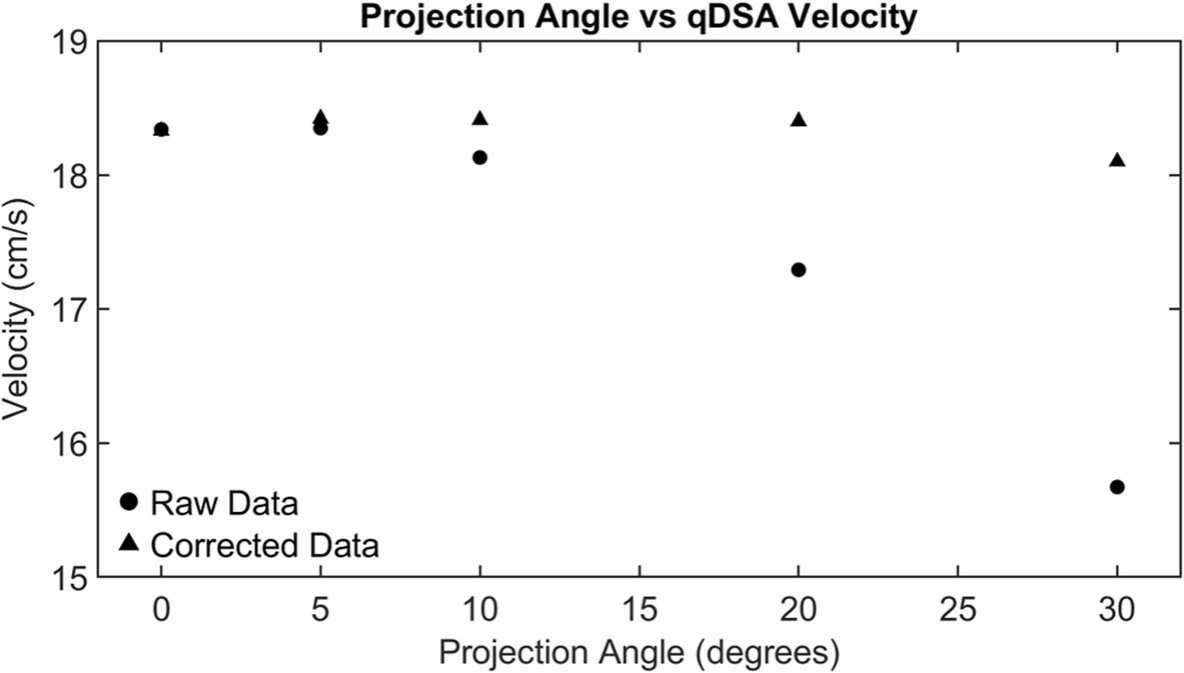
The raw qDSA velocity calculations as a function of projection angle are plotted as circles. As the projection angle increases the calculated velocity values decrease as a function of cosine the projection angle. Applying the projection angle correction reduces variation in the calculated velocity over a range of projection angles

**Fig. 4:**
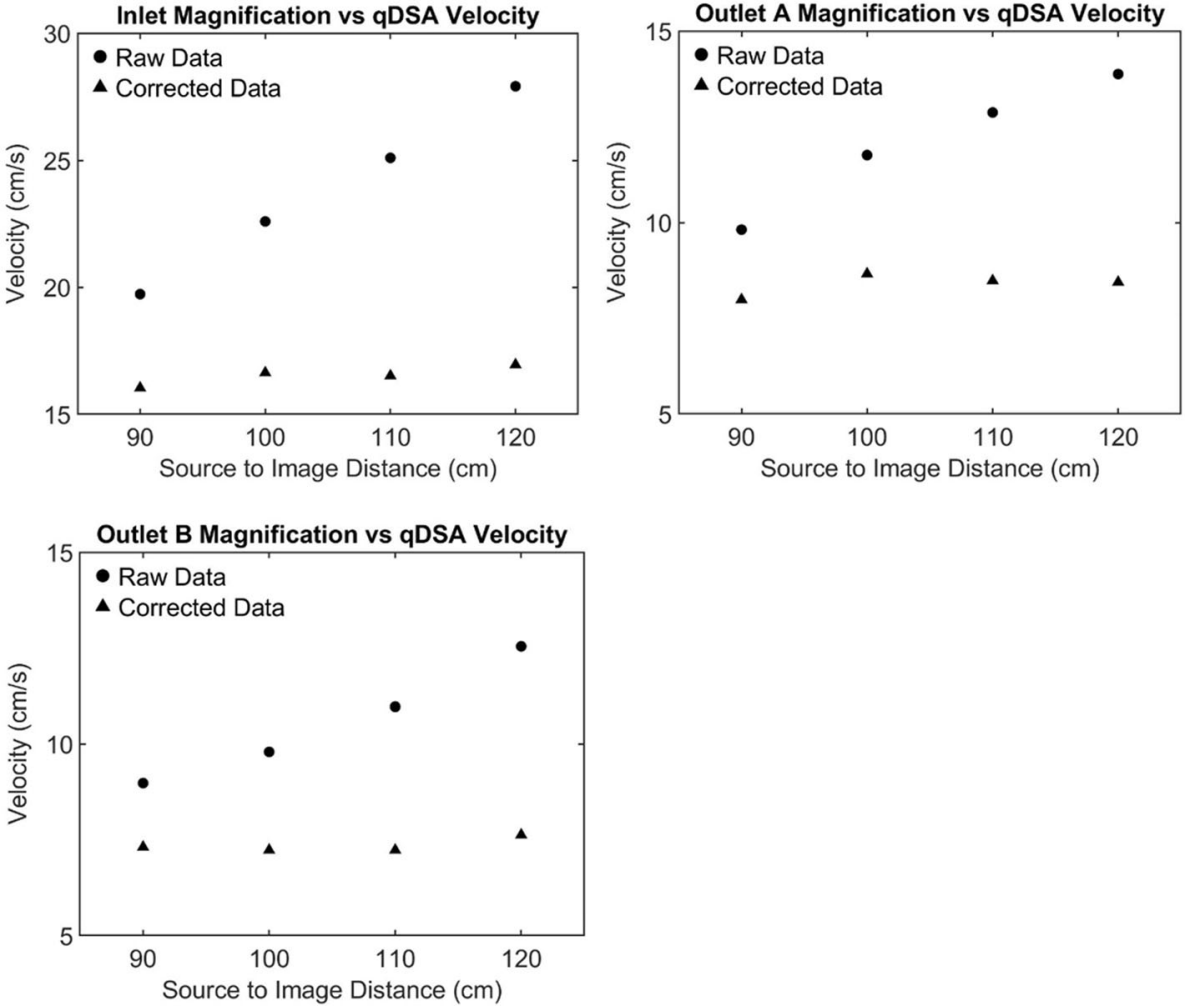
The raw qDSA velocity calculations as a function of source to image distance (SID) are plotted as circles. As the SID increases the magnification factor increases causing the calculated velocity values to increase as a function of magnification. Applying the magnification correction, using a reference object, reduces variation in the calculated velocity over a range of SIDs

The variance of qDSA velocities decreased after correction for magnification and projection angle. The flow probe’s baseline mean velocity (±SD) for projection angle and magnification was 14.18 ± 0.053 cm/s and 14.65 ± 0.129 cm/s respectively. The uncorrected and corrected velocities as a function of projection angle can be seen in Fig. 3. For projection angle correction, the qDSA variance (σ^2^ = 0.0745, 95% CI [0.0388,0.1933]) was less than the defined variance limit (5% Mean = 0.709) resulting in a failure to reject the null hypothesis (σ^2^ < 5% Mean, *p* = 0.999). The uncorrected and corrected velocities as a function of table height (magnification) for the inlet and outlet branches can be seen in Fig. 4. For magnification correction, the qDSA variance (σ^2^ = 0.2147, 95% CI [0.1073,0.6188]) was less than the defined variance limit (5% Mean = 0.733) resulting in a failure to reject the null hypothesis (σ^2^ < 5% Mean, *p* = 0.956).

### In vivo study

**Fig. 5:**
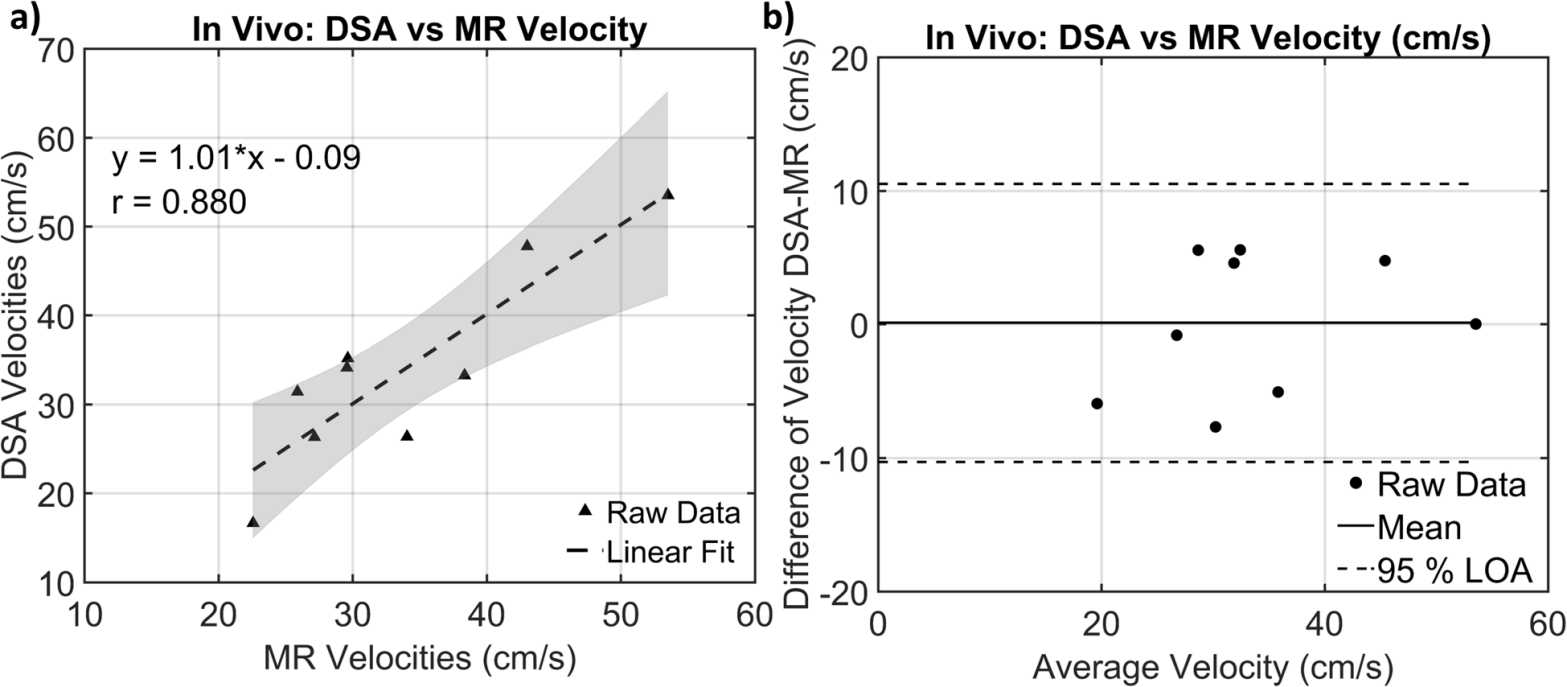
**a** Linear regression analysis between the 4D Flow MRI velocities and the calculated qDSA velocities (r = 0.880, p = <.001). The shaded region represents the 95% confidence band. **b** Bland-Altman analysis between the 4D Flow MRI and qDSA velocities (Bias = 0.117 cm/s, LOA [− 10.30 cm/s, 10.53 cm/s])

**Fig. 6:**
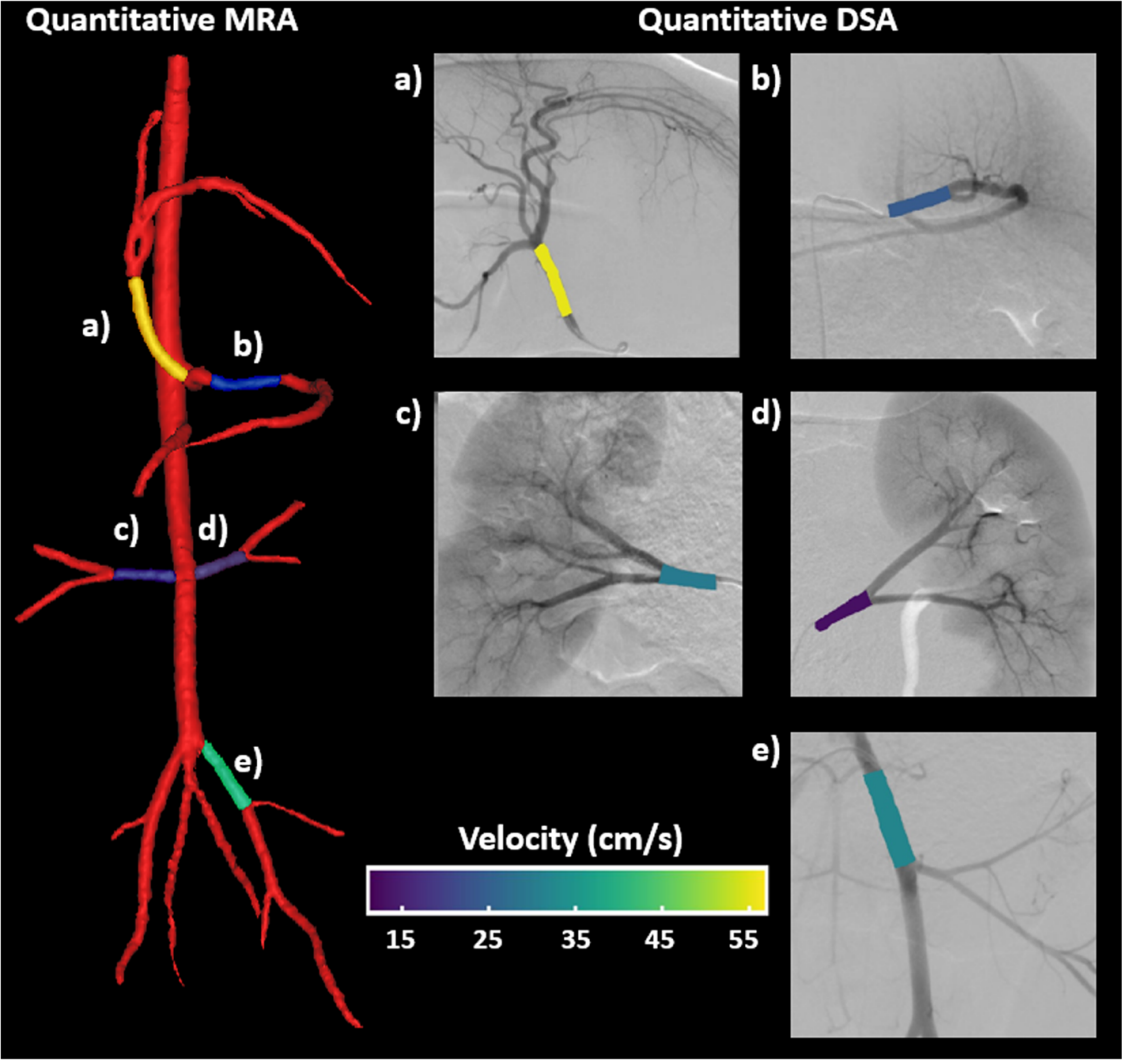
Quantitative angiographic images for both the 4D Flow MRI and qDSA methods from Swine 1. The magnetic resonance angiogram (MRA) 3D reconstruction was manually segmented and average velocities were calculated for the associated DSA vessels. The distribution of velocities was similar between both imaging modalities

Triplicate DSA acquisitions were successfully acquired at the five abdominopelvic arterial vessels of interest: left iliac, left renal, right renal, common hepatic, and splenic. Adequate downstream contrast mixing was seen in all in vivo DSA acquisitions, allowing for velocity calculations to be completed in the injected vessel. Linear regression between 4D flow MRI velocities and calculated qDSA velocities is shown in Fig. 5. The linear regression equation was: V_DSA_ = 1.01*V_MRI_ − 0.10, where V_DSA_ is the velocity calculated from qDSA and V_MRI_ is the velocity measured with 4D flow MRI. There was a strong correlation between velocity on 4D flow MRI and qDSA (r = 0.880, *p* < .01). The Bland-Altman analysis showed a bias of 0.117 cm/s between techniques with a upper limit of agreement of 10.53 cm/s and a lower limit of agreement of − 10.30 cm/s. Figure 6 shows the quantitative angiograms, color-coded by velocity, from the MRI and DSA scans from swine 1. The quantitative velocity values from MR and DSA for both swine can be found in Table 3. The distribution of velocities followed similar trends for both imaging modalities.

**Table 3:** Quantitative In Vivo Velocities

	MR Velocity (cm/s)	DSA Velocity (cm/s)	DSA Std. Dev.	Mag. Factor
**Swine 1**				
Common Hepatic	53.51	53.53	6.79	1.19
Splenic	27.15	26.33	4.53	1.11
Left Renal	22.57	16.65	1.98	1.28
Right Renal	25.88	31.44	4.39	1.16
Iliac	38.33	33.28	5.03	1.20
**Swine 2**				
Common Hepatic	43.01	47.78	8.78	1.28
Splenic	29.56	34.16	2.72	1.26
Right Renal	29.62	35.21	4.48	1.28
Iliac	34.05	26.38	3.11	1.33

Quantitative values from the in vivo study for both imaging modalities are presented in the table. The standard deviation of the DSA velocity is provided from the triplicate scans. The magnification factor was calculated for each vessel by imaging a reference wire, of 3 cm in length, in the vessel of interest prior to injection

## Discussion

This study investigated the feasibility of a quantitative angiography method using time-resolved 2D DSA. The method was assessed for accuracy and precision in a phantom model and an in vivo porcine model. Our results indicate that qDSA allows the calculation of quantitative velocities, over a range of physiologic abdominal arterial velocities (Nakamura et al. 1989) in near real-time, that are both accurate and precise. Potential errors from angle projection and magnification were investigated and successfully corrected for, demonstrating the robustness of the technique. Quantitative velocities were computed in vivo using branches of the abdominal aorta and were found to be strongly correlated with an established quantitative MRI technique. The application of the proposed method was similar for both the experimental and in vivo studies, indicating the potential for clinical adaptation.

Prior intraprocedural quantitative imaging techniques have been described, including both 2D and volumetric techniques (Shaughnessy et al. 2018; Wu et al. 2018; Wang et al. 2010; Zhang et al. 2013; Hinrichs et al. 2016; Wang et al. 2016). Volumetric techniques including quantitative 4D transcatheter intra-arterial perfusion (TRIP) MRI and 4D DSA have the ability to provide flow, not just velocity, which can be important for interventions in which vessel diameters change. TRIP MRI lacks feasibility as it requires a complex hybrid angiography/MR suite only available at select institutions, and also requires significant time and cost. 4D DSA is useful for characterizing blood flow at baseline and upon completion of a procedure, but the long data acquisition times and susceptibility to motion artifacts make it less suitable for repeated use throughout a procedure.

Quantitative color-coded DSA is a commercially available 2D DSA-based technique (syngo iFlow; Siemens, Forchheim, Germany). However, it does not provide true arterial velocity or flow, rather it provides time-attenuation curves for specified points and color-coded vessel displays based on TOA or TTP. Such an analysis is prone to error and sensitive to changes in cardiac output, motion, total amount and duration of contrast medium administration, imaging parameters (eg, injection delays), and angiographic catheter position. The proposed qDSA technique takes advantage of spatial and temporal information along the vessel allowing for a more robust quantitative technique. The ability to characterize intraprocedural arterial velocity reductions during TAE using qDSA was recently demonstrated in a clinically relevant porcine liver model (Periyasamy et al. 2020). In that study, qDSA was compared to the commercially available iFlow. qDSA was able to quantitatively discriminate between embolization endpoints including sub-stasis, the desirable clinical endpoint previously correlated with improved overall survival (Jin et al. 2011). qDSA better characterized blood flow changes when compared with iFlow and qDSA endpoints correlated with tissue level changes. The results of that study, which included an ability to resolve a range of changes in arterial velocity, support the potential clinical role for qDSA not only for TAE, but also for other arterial interventions (e.g. angioplasty or stenting for peripheral arterial disease) where accurate assessment of changes in blood velocity are critical in determining the success of a treatment.

The qDSA technique described here could be easily translated to intraprocedural clinical workflows given that it would only require modification to image acquisition parameters. In the present study, all velocity calculations were performed within 2–3 min of data acquisition using the prototype MATLAB tool on a standard laptop (Intel Core i7-8550U 1.80 GHz CPU, 16 GB RAM). Further refinement of the technique and tool will likely lead to significant reductions in computational times, enabling near real-time determination of velocities repeatedly during procedures. Although qDSA is currently limited to blood velocity, further development may allow for calculated velocities to be converted to blood flow using forward projection techniques on pre-procedure 3D imaging (Hentschke et al. 2011). This would permit flow quantification in a manner more similar to 4D Flow MR or 4D DSA.

An accurate velocity calculation requires precise knowledge of both the distance the blood traveled and the time (or temporal shift). The measured distance will differ from the true distance if the projection is not orthogonal to the vessel segment. The proper projection angle can be determined from 3D imaging, either preprocedure or at the time of the procedure, and the true distance can be determined by placing an object of known dimensions into the vessel segment (eg a guidewire or catheter with radiopaque markings). While both projection angle and magnification corrections are important to achieve accurate absolute velocity values, relative velocity changes during a procedure (eg, pre- and post-intervention) can be calculated without incorporating corrections as long as the table position and projection angle are maintained. Furthermore, corrections for projection angle may be unnecessary for many abdominopelvic interventions given that many vessels are relatively straight, vessels can typically be laid out in AP or shallow oblique projections, and minimal variation (< 5%) in velocity was observed over a wide range of projection angles (± 15 degrees) in the phantom study. A minimum frame rate is required to achieve adequate temporal resolution for the higher velocity values encountered in clinical practice. In its current form, qDSA is associated with additional radiation dose (from additional high frame-rate scans). Radiation dose reduction strategies are currently being explored to facilitate incorporation of qDSA into standard clinical angiography workflows. In addition, preliminary studies with both preclinical and clinical datasets indicate that qDSA may also be viable with fluoroscopy, which would significantly decrease the exposure compared to subtraction angiography.

X-ray videodensitometric blood velocity methods have been previously described (Shpilfoygel et al. 2000). Many of these blood velocity techniques were developed for cerebrovascular interventions. Few have been developed or validated in the abdominal vasculature. Furthermore, an analysis tool for near real-time calculation has yet to be created for use in body interventions. We have developed a tool that allows the calculation of blood velocity from 2D DSA sequences within minutes with minimal user interaction. The graphical user interface allows common visualizations of DICOMS and TACs while providing quantitative blood velocity values from temporally and spatially segmented vessels of interest. The development of this qDSA velocity tool makes intraprocedural blood velocity calculations feasible and more readily translatable to clinical workstations.

Our study had several limitations. Our preliminary in vitro testing was performed in a bifurcation phantom with three segments, all relatively linear and of constant diameter. The velocity in this phantom is primarily laminar, but the larger outlet does contain a region of recirculation. The abdominal arteries have more tortuosity and variation in vessel diameter which can lead to increased turbulent velocities and disruption in pulsatile signal. Additionally, we used a pump that generated a repeatable pulsatile signal, which may not entirely represent the hemodynamic heterogeneity of abdominal vasculature. Despite the limitations of the phantom model, the results of the in vivo experiments suggest the technique is robust and accurate with more complicated vessel geometries. In our in vivo testing, the MR velocity data was acquired without an intra-arterial injection requiring an additive velocity correction before a direct comparison to qDSA velocity could be made. To replicate the DSA injection during MRI, catheter placement could be completed under fluoro guidance and then the animal could be moved to the MRI scanner. However, for a multi-vessel analysis, this would require a minimum of 5 trips between imaging modalities with the risk of catheter movement occurring during each transport. Additionally, the MR scans would need to be adjusted to capture near real-time velocities, limiting the scans to 2D techniques which would no longer allow averaging along the vessels of interest.

## Conclusions

The proposed qDSA method allows for accurate and precise calculation of blood velocities, in near real-time, from time resolved 2D DSAs. Arterial blood velocity calculations on qDSA strongly correlated with established quantitative techniques. The major advantage of the proposed technique is near real-time measurement of relative and absolute changes in blood velocity. While our study focused on the accuracy, consistency, and feasibility of calculating blood velocity using time-resolved 2D DSA sequences, further investigation is necessary to evaluate the performance of the qDSA technique in an intra-procedural context.

## Supplementary Information

**Additional file 1.** Video demonstration of the qDSA tool.

## Data Availability

The datasets used for this study are large multidimensional datasets and may be made available from the corresponding author upon request.

## Abbreviations

TAE: Transarterial embolization
DSA: Digital subtraction angiography
TOA: Time of arrival
TTP: Time to peak
qDSA: Quantitative 2D digital subtraction angiography
TAC: Time-attenuation curve
CI: Confidence interval
